# Use of Fumed Silica Nanostructured Additives in Selective Laser Melting and Fabrication of Steel Matrix Nanocomposites

**DOI:** 10.3390/ma15051869

**Published:** 2022-03-02

**Authors:** Hwee Kang Koh, James Guo Sheng Moo, Swee Leong Sing, Wai Yee Yeong

**Affiliations:** 1 Singapore Centre for 3D Printing, School of Mechanical & Aerospace Engineering, Nanyang Technological University, 50 Nanyang Avenue, Singapore 639798, Singapore; m170010@e.ntu.edu.sg; 2 Evonik (SEA) Pte. Ltd., Asia Research Hub, 21 Biopolis Road, Singapore 138567, Singapore; james.moo@evonik.com; 3 Department of Mechanical Engineering, National University of Singapore, 9 Engineering Drive 1, Singapore 117575, Singapore

**Keywords:** additive manufacturing, 3D printing, selective laser melting, powder bed fusion, metal matrix composites

## Abstract

The advancement of additive manufacturing (AM) for metal matrix nanocomposites (MMNCs) is gaining enormous attention due to their potential improvement of physical and mechanical performance. When using nanostructured additives as reinforcements in 3D printed metal composites and with the aid of selective laser melting (SLM), the mechanical properties of the composites can be tailored. The nanostructured additive AEROSIL^®^ fumed silica is both cost-effective and beneficial in the production of MMNCs using SLM. In this study, both hydrophobic and hydrophilic fumed silicas were shown to successfully achieve homogenous blends with commercial 316L stainless steel powder. The powder blends, which exhibited better flow, were then used to fabricate samples using SLM. The samples’ microstructure demonstrated that smaller grains were present in the composites, resulting in improvements in mechanical properties by grain refinement compared to those of 316L stainless steel samples.

## 1. Introduction

Additive manufacturing (AM), also commonly known as 3D printing or formally known as rapid prototyping (RP), is a family of processes that produces parts by joining materials together, usually layer-by-layer, based on information retrieved from computer aided design (CAD) software without the use of physical moulds. They can be directly used to fabricate functional parts without the need for post-processing. AM also circumvents some of the limitations of conventional methods, such as the need for multiple exhaustive processes that must be used to produce sophisticated and complex parts [1].

AM has been increasingly used over the years and provides key benefits that product manufacturers over conventional manufacturing. These benefits include, but are not limited to, cost effectiveness from the short downtime in terms of prototyping, reduced material wastage, vast potential for product design innovation, and the ability to build complex workpieces which cannot be achieved through conventional processes [1]. AM is capable of fabricating parts using metals, ceramic, and polymers [2,3]. Among the many AM methods currently available in the industry, several processes such as selective laser melting (SLM) and direct metal laser sintering (DMLS) are used for the fabrication of metals. SLM and DMLS are also commonly called laser powder bed fusion (LPBF). Due to the inherent benefits of AM over conventional manufacturing, it has been adopted widely for manufacturing by many industries, including aerospace, medical, and automotive [4].

Stainless steel has been used widely across many industries including construction, automotive, and pharmaceutical. It has been one of the most essential and important materials to keep costs low compared to other available materials [5,6,7,8]. Among all grades of stainless steel, 316L stainless steel is commonly used due to its mechanical properties’ advantages, such as being highly resistant to corrosion and oxidation. Many papers in the literature reported on SLM 316L stainless steel samples with regard to the effect of processing parameters, such as laser power, scanning speed, hatch distance, and scanning pattern [5,6]. Many engineers have leveraged the advantages of this technology and have successfully experimented with adding in different kinds of additives to improve the characteristics of stainless steel by forming metal matrix composites (MMCs). MMCs have been effectively applied to aerospace, automotive, and medical industries, which require the latest material improvements [5]. MMCs are normally fabricated with a metallic matrix phase and with one or more different kinds of phases for reinforcement purposes. The reinforcements come in the form of particulates, whiskers, or fibres from materials such as ceramics, carbon or metallic materials. Studies have been conducted to improve the properties by introducing solid solution [9] and grain refinement [10]. Past research has involved adding in reinforcement such as silicon carbide (SiC), titanium diboride (TiB_2_), and titanium carbide (TiC) in 316L stainless steel to increase wear resistance [3,11,12]. Furthermore, investigations have also been conducted on the use of nanostructured materials such as silica, titania, carbon nanotubes, and layered silicates as reinforcements to modify the properties of different matrices [13,14]. There are reports regarding the use of small amounts of nanostructured materials to polymers, which was shown to improve the mechanical properties [15,16,17] and dimensional stability under creep conditions [18]. Among all the additives, fumed silica is a prime candidate that can be produced with different surface areas (50–400 m^2^/g) using a variety of surface treatments from hydrophilic to hydrophobic. These silica structures have a fractal structure, and can form a network of interconnecting particles [19].

The ability to be customised to suit the industry’s needs in comparison to conventional metals has increased the use of MMCs in the industry [20]. In the past twenty years, the use of MMCs has grown significantly [21]. Numerous researchers have conducted intensive tests and proven, with the aid of suitable reinforcement, that the shortcomings of properties in materials such as 316L stainless steel can be improved [11,22,23]. This led to an increase in demand for customised material, specifically with lightweight and high-performance parts, which has steered the advancement of metal matrix nanocomposites (MMNCs).

MMNCs, a version of MMCs, use nanostructured materials as reinforcements. Due to their nanostructure morphology, MMNCs are capable of performing better than MMCs for wear resistance, damping properties, and mechanical strength, which are key for various applications [24,25]. Experiments have been conducted and the results have shown that for aluminium matrix composites with only 3 vol % of Al_2_O_3_ nanostructured materials, the parts performed better in comparison to the same parts made of 10 vol % Al_2_O_3_ and 10 vol.% SiC reinforcement in the microscale [26,27].

Despite the exceptional gains in interest from these industries, the complex processes and lack of economic efficiency have restricted the use of MMNCs. However, SLM has become a viable processing technique due to its ability to fabricate near-net-shaped parts and recycle unused powder which lowered the cost of producing MMNCs. Thus, SLM shows rising popularity in the production of MMNCs with complex structures and properties at a fair cost [28,29,30,31]. This has led to various studies concentrating on the exploration and development of this process [32,33].

In this study, the properties of MMNCs fabricated under the same processing conditions with 316L stainless steel combined with reinforcement materials, which consisted of hydrophilic and hydrophobic fumed silicas from the same manufacturer, are analysed. The investigation also evaluate the compatibility of different reinforced materials in terms of distribution and agglomeration. Finally, conclusions were drawn on the effect of different properties of additives (hydrophilic or hydrophobic) on the MMNCs properties, fabricated using SLM by benchmarking them with 316L stainless steel.

## 2. Materials & Methods

### 2.1. Preparation of Powder

The 316L stainless steel powder used in this experiment was produced by TLS Technik Spezialpulver (Bitterfeld-Wolfen, Germany) and was spherical in shape with a particle size distribution between 20 and 63 µm. The two kinds of fumed silica powder used were AEROSIL^®^ R 812 S hydrophobic fumed silica and AEROSIL^®^ 200 hydrophilic fumed silica. AEROSIL^®^ 200 is a hydrophilic fumed silica with surface silanol groups; AEROSIL^®^ R 812 S is a hydrophobic fumed silica surface modified with hexamethyldisilazane. The same amount (0.05 wt %) of silica was added to the 316L stainless steel powder in this experiment to determine if one produced more advantages over the other by evaluating the characteristics of the specimens. The powders were mixed using an Inversina Tumbler Mixer (Bioengineering, Inc., Massachusetts, MA, United States). The mixing speed was set at 140 rpm and the duration was set at 4 h for the powder to flow and mix freely while using three-dimensional inversion kinematics, otherwise known as the Paul Schatz principle, to homogenize the powders. Powder flowability was measured using the ISO4490 standard with a Hall flowmeter (MZ-102, Mayzun, Shenzhen, China), which measures the time needed for 50 g of a sample to pass through a funnel with an aperture of 2.5 mm.

### 2.2. Selective Laser Melting

The SLM machine used to fabricate the specimens was an SLM 250HL (SLM Solutions AG, Lubeck, Germany). Its build volume can reach 250 × 250 × 250 mm^3^ and produce homogeneous metal components using fine metal powders. During the process of fabricating the specimens, argon gas was introduced into the system to prevent specimens from oxidising by keeping the oxygen level below 2%. During the process, a recoater was used to lay a coat of powder across the building platform. As it moved backwards, the recoater blade smoothed out the surface, and excess powder was collected in a container, which would be recycled. The laser beam scanned each layer of powder directed by the data of the stereolithography (STL) file. The building platform was lowered by the layer thickness defined after each scan before a new coat of powder was laid by the recoater. This cycle repeated until the final parts were fabricated. The processing parameters used for fabricating the specimens were laser power of 400 W, scanning speed of 760 mm/s, hatch spacing of 0.120 mm, and layer thickness of 0.05 mm. The stripes laser scanning pattern was used.

A total of three sets of five cubic specimens, with each set for 316L stainless steel, 316L stainless steel with AEROSIL^®^ R 812 S, and 316L stainless steel with AEROSIL^®^ 200, were fabricated for testing and evaluation. Each cube had dimensions of 10 × 10 × 10 mm^3^ and were fabricated to conduct density, surface roughness, microhardness tests and microstructure analysis.

### 2.3. Density

The Archimedes method was used to measure the density of the specimens. The measurements were taken using a XS204 density test kit (Mettler Toledo, Columbus, OH, USA), with 99.9% ethanol at 23.8 °C. Five replicates for each material were used in the density measurement.

### 2.4. Surface Roughness

The surface roughness of the samples was measured using a confocal microscope (VK-X130K, Keyence Corporation, Osaka, Japan) to find the roughness average (Ra). Five replicates were used in the measurement with five Ra values obtained for each surface. Each specimen’s measurement was taken straight after removal from the substrate to evaluate the surface produced by SLM and to identify any characteristic changes due to the different additives.

### 2.5. Grinding and Polishing

After the samples were mounted, grinding and polishing were performed prior to performing microhardness and microstructure tests. The samples were first processed on a grinding and polishing machine (Tegramin-25, Struers, Copenhagen, Denmark) with 320 grit SiC paper for 5 min, followed by MD-Largo and DiaPro Largo 9 μm suspension for 5 min. The specimens then underwent polishing steps with MD-Dac and DiaPro Dac 3 μm suspension for 5 min and another polish with MD-Nap and DiaPro Nap 1 μm suspension for 3 min. Lastly, in order to achieve a mirror finish for the samples, they were polished with Chem and OP-S 0.25 μm suspension for 1 min. All the polishing cloths and suspensions are from Struers (Copenhagen, Denmark). All grinding and polishing steps were completed with 150 rpm co-rotation and 25 N force pressing on the samples.

### 2.6. Microstructure Analysis

Microstructure analysis was carried out on the mirror polished and etched surfaces (using etchant containing HCl and HNO_3_ in a ratio of 1:1). Microstructural analysis was also performed on the powders of 316L stainless steel, 316L stainless steel with AEROSIL^®^ R 812 S and 316L stainless steel with AEROSIL^®^ 200. All (5 replicates) top and side surfaces of the cubes and fractured surfaces of the tensile test coupons were also evaluated.

### 2.7. Microhardness Testing

The Vickers microhardness testing was performed using an FM-300E microhardness tester (Future-Tech Corp., Kanagawa, Japan). Five replicates were used, with 10 measurements obtained from each top and side surface.

### 2.8. Tensile Test

Tensile test coupons were fabricated to evaluate the performance of 316L stainless steel, 316L stainless steel with AEROSIL^®^ R 812 S, and 316L stainless steel with AEROSIL^®^ 200. Five test coupons following the ASTM E8/E8M standard for the tensile testing of each material were fabricated. The dimensions of the samples followed the subsize specimen stated in the standard, with a gauge length of 40 mm. The tensile tests were conducted using an Instron Static Tester Series 5569 (Instron, Norwood, MA, USA) with a 50 kN load cell and a strain rate of 1 mm/min.

## 3. Results and Discussion

**Figure 1:**
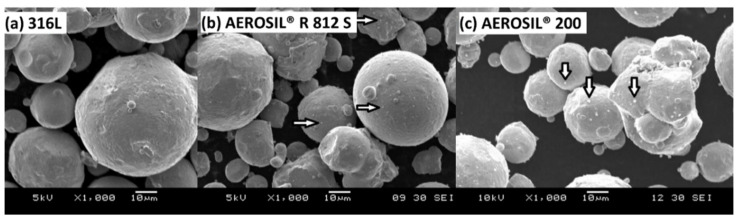
Scanning electron microscopy of (**a**) 316L stainless steel, (**b**) 316L stainless steel with 0.05 wt % AEROSIL^®^ R 812 S, and (**c**) 316L stainless steel with 0.05 wt % AEROSIL^®^ 200 powder at 1000× magnification.

Fumed silica products of AEROSIL^®^ were mixed homogenously with 316L stainless steel powders. The two additives were homogenously coated onto the 316L stainless steel powder particles, as shown in Figure 1. The nanostructure fumed silica is shown as a white spot that spread evenly on 316L stainless steel particles, indicated by the white arrows.

**Figure 2:**
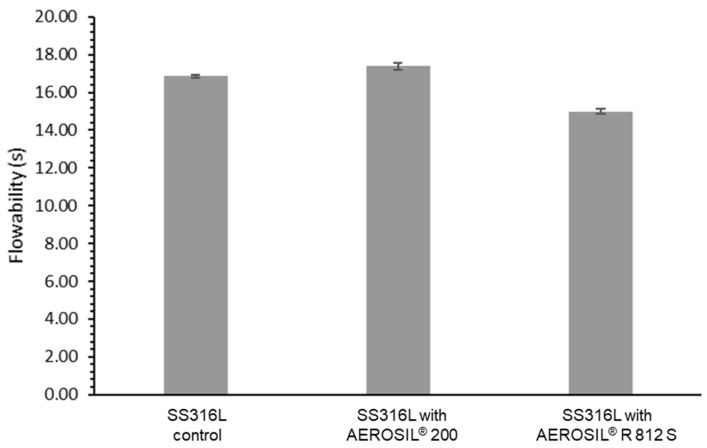
Flowability of 316L stainless steel, 316L stainless steel with 0.05 wt % AEROSIL^®^ 200, and 316L stainless steel with 0.05 wt % AEROSIL^®^ R 812 S powders.

In Figure 2, the addition of 0.05 wt % AEROSIL^®^ 200 or AEROSIL^®^ R 812 S had a slight effect on the flowability of the powders. This is attributed to the fact that the 316L stainless steel powder particles were already spherical, hence possessed adequate flowability. The total flow time taken for a 50 g sample was used as a gauge for flowability using a Hall flowmeter. There was little improvement in the metal powder’s flow, with a total flow time of 16.8 s for 316L stainless steel powder compared to 17.2 s with the addition of 0.05 wt % AEROSIL^®^ 200. However, a reduction to a flow time of 14.8 s with the addition of 0.05 wt % AEROSIL^®^ R 812 S was observed, corresponding to a 12% improvement. The hydrophobic modification of the silica surface in AEROSIL^®^ R 812 S fumed silica particles reduced cohesive forces between the powder particles, enabling the silica particles to serve as spacers to improve powder flow.

The density of the SLM fabricated samples is shown in Table 1. With a small amount of additives added, there was an insignificant change in the densities in terms of average density, which was expected. The small amount of additives was not effective in modifying the thermal properties of the powder. However, AEROSIL^®^ 200 significantly lowered the density’s standard deviation, which may be attributed to more stable melt flows during SLM. A higher stability is desirable in terms of fabrication. Smaller deviations in density refer to the ability to produce consistent parts, which can eventually reduce quality control issued and downtime.

In this experiment, marginal improvement in the surface roughness was observed in the specimens mixed with AEROSIL^®^ R 812 S in terms of average value and smaller standard deviation compared to that of 316L stainless steel. The average value and standard deviation taken from the samples mixed with AEROSIL^®^ 200 showed a slightly rough texture, as shown in Table 2. The surface roughness of the samples could have been due to the partially melted particles adhering to the specimens.

**Figure 3:**
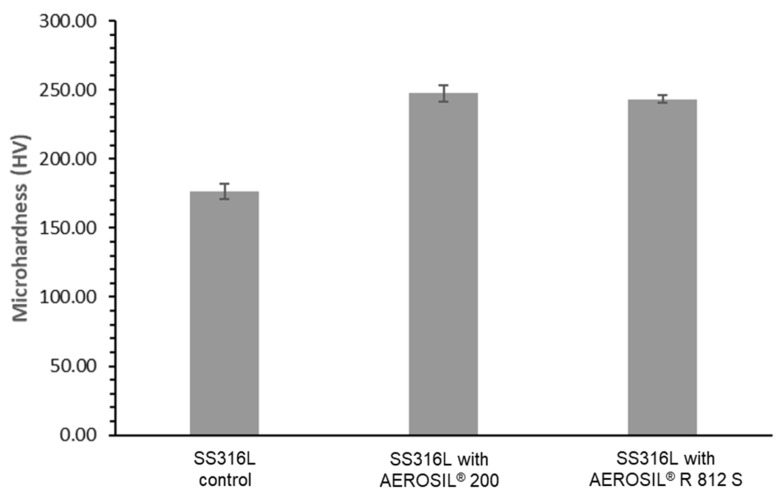
Microhardness of SS316L, SS316L with 0.05 wt % AEROSIL^®^ 200, and SS316L with 0.05 wt % AEROSIL^®^ R 812 S.

Microhardness tests were performed to investigate the surface hardness of the produced samples. The results shown in Figure 3 show improvement in the microhardness of specimens with the addition of 0.05 wt % AEROSIL^®^ 200 and 0.05 wt % AEROSIL^®^ R 812 S. This shows that there was a significant improvement in the microhardness of the parts manufactured with silica additives. The microhardness of the 316L stainless steel, 316L stainless steel with AEROSIL^®^ 200, and 316L stainless steel with AEROSIL^®^ R 812 S are 177, 247, and 242 HV, respectively. As such, 40% and 37% improvements due to the silica additives AEROSIL^®^ 200 and AEROSIL^®^ R 812 S were achieved, respectively. Such improvements present an opportunity for enhancing the wear and durability of functional parts. The low standard deviation showed that the hardness of the specimen was relatively consistent throughout the surface, which is desirable. These consistent hardness values lead to accurate material selection, which might have otherwise resulted in premature mechanical failure.

**Figure 4:**
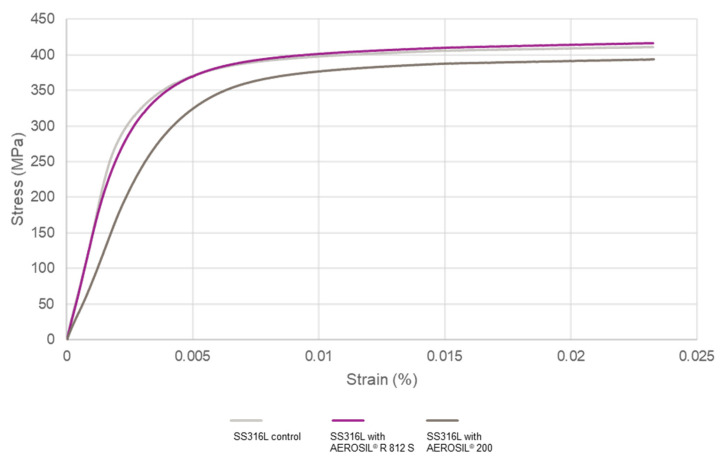
Tensile properties of 316L stainless steel, 316L stainless steel with 0.05 wt % hydrophobic AEROSIL^®^ R 812 S, and 316L stainless steel with 0.05 wt % hydrophilic AEROSIL^®^ 200.

A comparison of the tensile properties of 316L stainless steel and 316L stainless steel with AEROSIL^®^ R 812 S and AEROSIL^®^ 200 is shown in Figure 4 and Table 3. The specimens with AEROSIL^®^ R 812 S additives had properties that were comparable to those of 316L stainless steel, while specimens with AEROSIL^®^ 200 showed poorer performance compared to those with AEROSIL^®^ R 812 S additives, but they still performed better than the 316L stainless steel samples. The test results demonstrated that the MMNCs produced have better tensile properties than 316L stainless steel.

**Figure 5:**
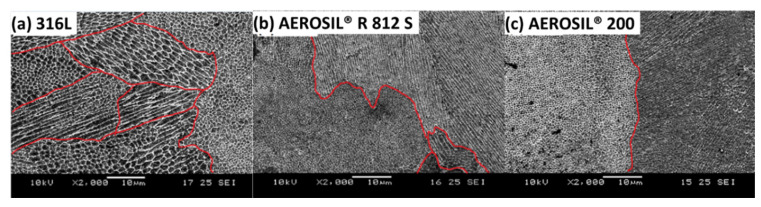
Scanning electron micrograph of (**a**) 316L stainless steel, (**b**) 316L stainless steel with 0.05 wt % AEROSIL^®^ R 812 S, and (**c**) 316L stainless steel with 0.05 wt % AEROSIL^®^ 200 after etching in 2000× magnification.

As stated in Table 3, 316L stainless steel with AEROSIL^®^ R 812 S had better ultimate tensile strength at 495 MPa than 316L stainless steel with AEROSIL^®^ 200 at 485 MPa and 316L stainless steel at 492 MPa, while maintaining a satisfactory Young’s modulus, yield strength, and elongation. However, SS316L with AEROSIL^®^ 200 suffered from a reduction in Young’s modulus to 82 GPa and a yield strength of 284 MPa compared to the control of SS316L at 148 GPa and 363 MPa, which are reductions of 44% and 22%, respectively. This phenomenon is explained later using Figure 5.

The differences in tensile properties between the samples can be explained by the differences in their microstructure. Samples with columnar grains have lower strength but higher plasticity, while those with equiaxed grains have higher strength and lower plasticity. A mix of equiaxed grains and columnar grains and, to an extent, an even distribution of the two grain types, would provide the optimal balance of strength and plasticity [34]. Specimens of 316L stainless steel with AEROSIL^®^ 200 had a more distinct segregation of equiaxed grains and columnar grains compared to pure 316L stainless steel specimens, as shown in Figure 5. The distinct segregation could be a reason for the reduction in the mechanical properties of the specimens of 316L stainless steel with AEROSIL^®^ 200.

**Figure 6:**
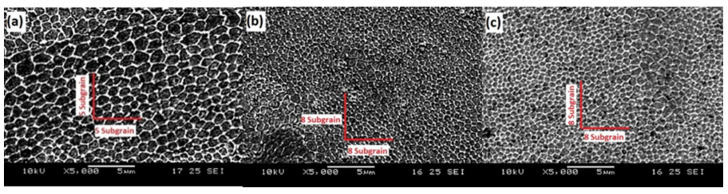
Scanning electron micrograph of (**a**) 316L stainless steel, (**b**) 316L stainless steel with 0.05 wt % AEROSIL^®^ R 812 S, and (**c**) 316L stainless steel with 0.05 wt % AEROSIL^®^ 200 after etching in 5000× magnification.

Another significant difference worth mentioning is the grain size observed in Figure 6. Based on image measurements, the 316L stainless steel specimens contained five grains within 5 μm, while both 316L stainless steel with AEROSIL^®^ R 812 S and 316L stainless steel with AEROSIL^®^ 200 specimens showed eight grains in 5 μm. This implies that the additives resulted in smaller grains, which likely led to the improvements in microhardness observed in Figure 2. The increase in their mechanical properties is due to the reduction in grain size, which potentially leads to improved microhardness of the specimens when accompanied by ideal grain segregations [34].

## 4. Conclusions

In this investigation, adding fumed silica to 316L stainless steel showed the potential to improve the mechanical properties of parts fabricated by SLM. This was achieved by using additives to manipulate the microstructure of the MMNCs, which resulted in grain refinement and distinct segregation between the equiaxed and columnar grain structures. The 316L stainless steel with hydrophobic AEROSIL^®^ R 812 S fumed silica specimens had lower surface roughness and improved microhardness. Furthermore, the 316L stainless steel with AEROSIL^®^ R 812 S specimens achieved tensile properties comparable to those of 316L stainless steel. This shows that the mechanical properties of the material can be improved with the introduction of fumed silica as nanostructured reinforcements in MMNCs. However, the 316L stainless steel with hydrophilic AEROSIL^®^ 200 fumed silica specimens performed poorly compared to 316L stainless steel in several aspects and, thus, may not be a suitable additive for MMNCs produced by SLM. Future work can consider adding different weightages of fumed silica to investigate and understand the relationship with regard to the changes in material characteristics.

## Figures and Tables

**Table 1:** Density of SLM specimens.

Sample	SS316L	AEROSIL^®^ R 812 S	AEROSIL^®^ 200
Density (g/cm^3^)	7.722 ± 0.012	7.718 ± 0.011	7.718 ± 0.005

**Table 2:** Surface roughness of SLM specimens.

Sample	SS316L		SS316L with AEROSIL^®^ R 812 S		SS316L with AEROSIL^®^ 200	
Surface	Top	Side	Top	Side	Top	Side
Ra (µm)	15.6 ± 1.5	16.6 ± 1.4	14.0 ± 1.7	13.8 ± 0.7	17.6 ± 7.0	15 ± 1.5

**Table 3:** Young’s modulus, ultimate tensile strength, yield strength, and elongation of SLM-built samples.

Specimen	SS316L	SS316L with AEROSIL^®^ R 812 S	SS316L with AEROSIL^®^ 200
Young’s modulus (GPa)	147.91 ± 5.67	143.14 ± 8.71	81.84 ± 6.25
Ultimate tensile strength (MPa)	491.76 ± 11.05	494.78 ±16.54	485.05 ± 14.56
Yield strength (MPa)	363.4 ± 8.22	358.9 ± 12.63	283.7 ± 10.29
Elongation (%)	38.14 ± 3.86	35.18 ± 5.23	37.47 ± 2.71

## Data Availability

Data are made available upon request, subjected to confidentiality clauses.
